# The Complete Nucleotide Sequence of Barley Yellow Dwarf Virus-PAV from Wheat in Turkey

**DOI:** 10.1128/mra.00745-22

**Published:** 2022-09-26

**Authors:** Havva Ilbağı, Rick E. Masonbrink, W. Allen Miller

**Affiliations:** a Department of Plant Protection, Agricultural Faculty, Tekirdağ Namık Kemal University, Tekirdağ, Turkey; b Genome Informatics Facility, Iowa State University, Ames, IA, USA; c Department of Plant Pathology, Entomology and Microbiology, Iowa State University, Ames, IA, USA; Portland State University

## Abstract

We report the sequence of an assembled genome of *Barley yellow dwarf virus-PAV* (BYDV-PAV) from Turkey. This 5,672 nucleotide RNA encodes seven known open reading frames and a possible eighth. This genome from wheat is closely related to BYDV-PAVs in Pakistan, Brazil, and Australia, including one sequenced 34 years ago.

## ANNOUNCEMENT

Yellow dwarf viruses (YDVs) in the *Luteovirus* and *Polerovirus* genera cause substantial yield and quality losses in small grain cereals worldwide (1–4). BYDV-PAV is the most common species of YDV, and thus, is of particular interest. Its genomic RNA encodes ORFs 1, 2, 3, 3a, 4, 5, and 6, which are translated via noncanonical mechanisms (4). The viral RNA has neither a 5’ cap nor a poly(A) tail (4).

**FIG 1:**
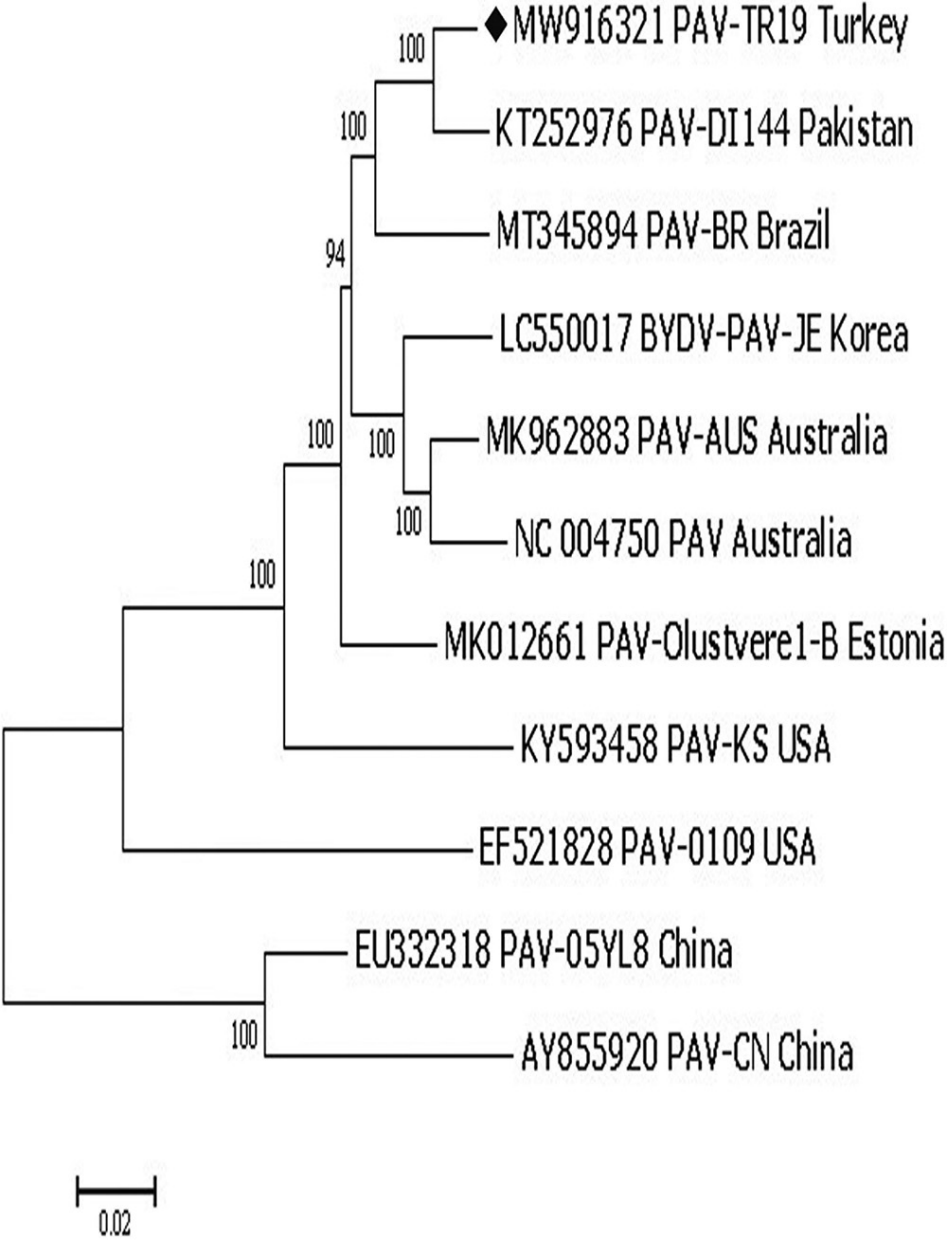
Phylogenetic tree constructed using the neighbor-joining method based on alignment of complete genome sequences of *Barley yellow dwarf virus-PAV* with the indicated GenBank accession numbers. 1,000 bootstrap replicates were used. Solid diamond indicates the sequence presented here.

A wheat plant exhibiting yellowing and dwarfing signs was collected from Trakya (European) region (41°0.02'25.6 N, 27°0.39'13.1 E) of Turkey in 2018. Total RNA was extracted from 100 mg leaf tissue using Direct-zol RNA miniprep kit (Zymo Research, Irvine, CA), followed by rRNA depletion (RiboZero, Illumina). A library was then generated with an NEBNext RNA Library Prep Kit (New England Biolabs, Ipswich, MA) and sequenced using paired end reads at 150 nucleotides each on a Hiseq 3000 system (Illumina Inc., CA). BBTools (5) was used to remove the adapter and barcode sequences from raw reads and to filter out low-quality reads (cutoff Q = 26). The processed reads were assembled *de novo* using the SPAdes v 3.15.1 tool (6), then annotated with NCBI Blast (7) against ViralDb (updated February 2021) within a threshold of percentage identity 50 and E value <10^−5^. The library had 17,749,192 paired end reads; 5,116 of these reads aligned to an in-house BYDV-PAV assembly using Hisat2 2.2.0, achieving 135.2 coverage. The resulting genome assembly, which we call BYDV-PAV-TR19 (GenBank number MW916321), has a complete length of 5,672 nucleotides and GC content of 49.1%. We used BLASTn 2.11.0 to compare the sequence to ten BYDV genomes from five continents: KT252976, MT345894.1, MK012661.1, MK962883.1, NC_004750.1, KY593458.1, EF521828.1, EU332318.1, LC550017, and AY855920.1. The BYDV-PAV-TR19 genome fits the highly conserved BYDV-PAV consensus at its 3’ terminus, CACUCGAAAGAGCAGUUCGGCAACCC, and at the 5’ end, AGUGAAGAUUGACCAUCU, except BYDV-PAV-TR19 starts with a C instead of an A at its 5’ end (although, for many isolates this 5’-terminal base is unknown). This indicates that MW916321 is almost certainly the complete genome sequence of BYDV-PAV-TR19. This sequence has the highest identity to sequence (KT252976) 1 at 97.4% across 5,659 nucleotides. The 5’ UTR of MW916321 is 141 nt long, while the 3’ UTR is 627 nt long, or 201 nt if ORF7 is translated. This potential novel ORF is not present in all BYDV-PAV sequences. Obtained nucleotide and deduced amino acid sequences were aligned with Bioedit (version 7.2.5). The alignments of complete nucleotide sequences were used to construct phylogenetic trees with the neighbor-joining distance method in Mega 7 (8) (Fig. 1). Complete genome sequence comparisons (Table 1; Fig. 1) indicate the highest nucleotide and amino acid sequence identities with BYDV-PAV–DI114 from Pakistan at 97% to 86%, respectively. Chinese isolates BYDV-PAV-CN and BYDV-PAV-05YL8 were the most distantly related isolates (Table 1).

**TABLE 1:** Percentage of overall genome nucleotide (above the diagonal) and concatenated amino acid (below the diagonal) sequence identities between BYDV-PAV-TR19 isolate and other BYDV-PAV sequences indicated with GenBank accession numbers

Location	Turkey	Pakistan	Brazil	Kansas	Estonia	China	Australia	China	Iowa	Australia	South Korea
Isolate accession no.	PAV-TR19	PAV- DI144	PAV-BR	PAV-KS	PAV-K Olustvere1-B	PAV-05YL8	PAV-AUS	PAV-CN	PAV-0109	PAV	PAV-JE
PAV-TR19 MW916321		**97** ^*a*^	94	89	94	80	94	**76** ^*a*^	84	93	93
PAV-DI144 KT252976	**86** ^*a*^		94	88	93	80	93	76	84	92	92
PAV-BR MT345894	81	87		88	94	80	93	77	84	93	92
PAV-KS KY593458	73	74	74		89	82	89	79	80	88	89
PAV-Olustvere1-B MK012661	80	77	78	74		80	93	76	85	92	94
PAV-05YL8 EU332318	55	55	56	72	56		81	92	79	80	80
PAV-AUS MK962883	83	79	79	51	78	56		77	83	97	96
PAV-CN AY855920	**50** ^*a*^	50	50	51	49	74	50		78	77	76
PAV-0109 EF521828	62	68	68	58	64	53	61	52		82	84
PAV NC_004750	85	77	77	72	76	56	91	50	60		94
PAV-JE LC550017	80	76	77	72	85	57	84	49	61	80	

a Bold, underlined text indicates highest and lowest sequence identities.

### Data availability.

The complete genome sequence of the BYDV-PAV-TR19 is in GenBank under accession number MW916321 and SRA accession number SRS5023133.
